# Mitigating climate change impacts on health: a comparative analysis of strategies in Saudi Arabia and Lebanon

**DOI:** 10.3389/fpubh.2025.1551559

**Published:** 2025-04-28

**Authors:** Alissar Al Khatib, Bshayer Alsaleh, Mohammed Almari, Salwa Hassanein, Nawal Al Hakawati

**Affiliations:** ^1^Department of General Studies, Almoosa College of Health Sciences, Al Ahsa, Saudi Arabia; ^2^Department of Nursing, Almoosa College of Health Sciences, Al Ahsa, Saudi Arabia; ^3^Department of Community Health Nursing, Cairo University, Cairo, Egypt; ^4^Department of Biological Sciences, Faculty of Science, Beirut Arab University, Tripoli, Lebanon

**Keywords:** climate change, mitigation strategies, Saudi Arabia, Lebanon, health impacts

## Abstract

**Introduction:**

Human activities are now adding rapidly more greenhouse gases to the atmosphere causing global warming which is one aspect of climate change, the greatest threat to public health. Therefore, this study aims to compare the health impacts of climate change on Saudi Arabia and Lebanon, and assessing their adaptation strategies in addressing climate change challenges.

**Methodology:**

This study is a descriptive Comparative Analysis, this was performed by analyzing the available data on climate-related health outcomes: food insecurity, emergence of infectious disease and car accidents and by comparing trends and percentages between the two countries.

**Results and discussion:**

Saudi Arabia and Lebanon has markable high CO_2_ emission, which negatively affect the health of people such as Food insecurity (in KSA: The estimated loss over the periods in all the crops ranges from 7 to 25%, in Lebanon: There is a decreased the growth of coveted crops, and increased the growth of weeds and pests), Road traffic accidents (approximately 1.3 million people die as a result of road traffic accidents and 20–50 million people suffer from other injuries.), and Emergence of infectious diseases (in KSA: an increase in 1°C of temperature caused a significant increase (15–25%) in malaria incidence, and increase in risk of food- borne diseases, in Lebanon: There is a vulnerability to the rise in food-borne and vector-borne diseases.). Forecasting the future for both countries reveal to a definite climate change occurring. Further actions could be implemented to overcome the negative health outcomes according to each country. Agriculture and Food Security, Use of renewable energy, and Awareness Campaigns on climate change and health are measures that could be implemented to face the outcomes of climate change. Interestingly, there are some organizations funding initiatives and activities in raising awareness of climate change.

**Conclusion:**

Numerous sectors are impacted by climate change, which is a serious issue that requires immediate action. It has a substantial influence on many different sectors and leads to food instability, agricultural issues, an increase in infectious disease transmission, and a rise in traffic accidents. These elements require particular care, and appropriate action should be done to eliminate them.

## 1 Introduction

**Figure 1:**
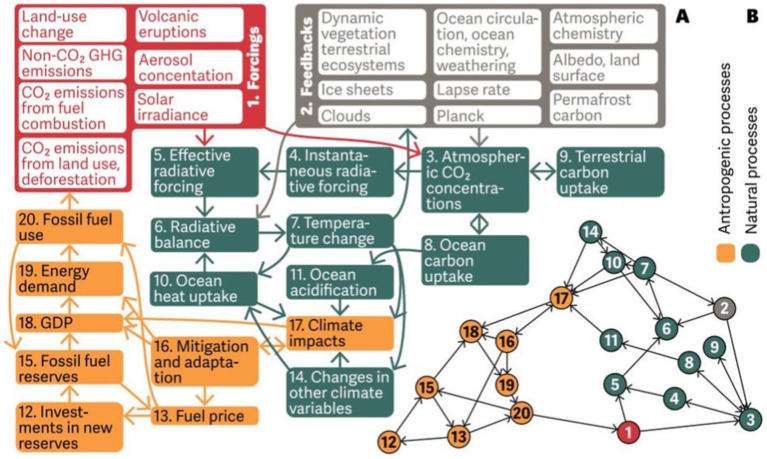
A climate model referenced by Holme and Rocha (89). Source: Knutti and Rugenstein (90).

Greenhouse gases have always occurred naturally in the atmosphere. These gases are water vapor, carbon dioxide, methane and nitrous oxide (1). Greenhouse effect occurs when greenhouse gases trapped the energy in the atmosphere and reflected back to the Earth surface, heating the planet even more. Having a small greenhouse effect is good and keeps Earth planet warm and habitable. The large naturally occurring carbon dioxide sinks such as forests and oceans have absorbed a large amount of greenhouse gases and maintained a relatively stable planet in terms of greenhouse effect (2). However, human activities including burning fossil fuels (Figure 1), intensive farming and agriculture and deforestation, are now adding rapidly more greenhouse gases to the atmosphere (3). As human activity expedites the concentration of greenhouse gases (GHG) in the atmosphere, the percentage of gases, carbon dioxide, methane and nitrous oxide has been rising rapidly. On the other hand, the human activities emitted other potent greenhouse gases such the Fluorinated ones. Most Fluorinated gases have high stability in the atmosphere with lifetime of 50,000 years with 23,000 times greater global warming potential than greenhouse CO2 (4).

Also, there has been an increase of CO_2_ emissions since 1850 (5), causing global warming, which is one aspect of climate change, the greatest threat to public health (6, 7). Consequently, the Earth’s global average surface temperature has been rising till this day. According to Bongioanni et al. (8), the Earth’s average surface temperature is 1.3°C warmer as of 2020 where this value is expected to increase with time., rendering the global warming a direct cause of climate change which is one of the most daunting issues the world is experiencing today (9, 10).

The elevated temperatures lead to dire consequences on the environment including elevated ocean temperatures, melting glaciers, and rising sea levels. Also, natural disasters like droughts, wildfires, floods, and hurricanes occurred more often, as cited by Akaev and Davydova (11). According to WHO, an increase of 250,000 extra deaths will be recorded between 2030 and 2050, that this is due to climate change (12). It is estimated that 38,000 of those deaths would be older adult people unable to withstand the prolonged heat exposure to high temperatures. This exposure could also lead to the worsening of cardiovascular (13) and neurological (14) diseases. Moreover, a systematic review done by Rocque et al. (15), found a correlation between cardiovascular and respiratory disease and droughts which is one of the increasingly occurring natural disasters as a result of global warming (16). In addition to that, storms might carry sea water to land which leads to microorganisms like *Vibrio* surfacing the land and inducing foodborne illnesses or diseases spread by wound exposure (17). Climate change is also directly linked to air pollution where the high temperatures correlate to an increase of ozone concentrations (18). The pollution causes damages to vital organs causing health complications like stroke, heart disease, lung cancer, asthma, chronic obstructive pulmonary disease, and respiratory infections which results in premature deaths ((19, 20), and (21)). According to the WHO (22), about 7 million deaths are caused by air pollution including 4.2 million pediatric deaths. In Mediterranean region, the more vulnerable communities, especially those with chronic disease will be at a higher risk of food insecurity, accelerating harmful consequences on productivity, increasing the rate of migration, nutrition and health (23) Therefore, this study aims to compare the health impacts of climate change on Saudi Arabia and Lebanon, and assessing their adaptation strategies in addressing climate change challenges.

### 1.1 Research questions

1. What is the main health impact of climate change in Saudi Arabia compared to Lebanon?
2. What are the predicted future adverse impacts of climate change on health in Saudi Arabia and Lebanon?
3. What are proactive measures implemented in Saudi Arabia and Lebanon to mitigate climate change in terms of funding sources and policy recommendations?

## 2 Methodology

### 2.1 Study design type

This study is a descriptive Comparative Analysis.

### 2.2 Study objective

This study aims to compare and describe the health impacts of climate change in Saudi Arabia and Lebanon. This was performed by analyzing the available data on climate-related health outcomes such as food insecurity, emergence of infectious disease and car accidents and by comparing trends and percentages between the two countries.

### 2.3 Data collection

Data Sources:

- Reports issued by World Health Organization (WHO), United Nations (UN), United Nations Framework Convention on Climate Change (UNFCCC) and World Bank Climate Change Knowledge Portal on the health impact of climate change in Saudi Arabia and Lebanon.
- Peer-Reviewed papers: published articles related to the impact of climate change on health in Saudi Arabia and Lebanon.
- National Health Records: Official national health data related to climate sensitive water-, vector-and food borne diseases, rate of care accident in addition to percentage of food insecure population both countries.
- Climate Data: National meteorological and climate reports to showing the variation of the temperature in both countries in addition to temperature increase estimation and projection for the coming years.
- Time Period: Available Data from the last 10 years.

### 2.4 Inclusion and exclusion criteria

#### 2.4.1 Inclusion criteria

Reports, records published papers addressing the impact of climate change on health in Saudi Arabia and Lebanon including data from:

- Household Food Insecurity Access Scale (HFIAS)
- Total number of injuries (TIJ) and total number of deaths due to road accidents
- Percentage and rate of climate change related diseases such as malaria

#### 2.4.2 Exclusion criteria

- Studies unrelated to climate change or health impacts
- Studies unrelated to Saudi Arabia and Lebanon

### 2.5 Data analysis

A descriptive analysis was conducted in this study in terms of

- Climate and location Data Comparison
- Health Impact Indicators
- Health Data Comparison: including quantitative data and trend analysis of
  - Food insecurity due to loss of crops yields and inaccessibility of water
  - Road accidents
  - Emergence of infectious disease

### 2.6 Mitigations and public health measures comparison

To evaluate and compare the mitigation strategies and public health responses to climate change in Saudi Arabia and Lebanon, this study adopted a qualitative comparative analysis (QCA) approach. This involved a review of national policies, climate adaptation plans, and health sector strategies.

Key dimensions for comparison included:

- Agriculture and food security management
- Use of renewable energy
- Awareness campaigns on climate change and health

## 3 Findings and discussion

### 3.1 Climate change challenges

#### 3.1.1 Saudi Arabia and Lebanon’s climate change challenges

**Figure 2:**
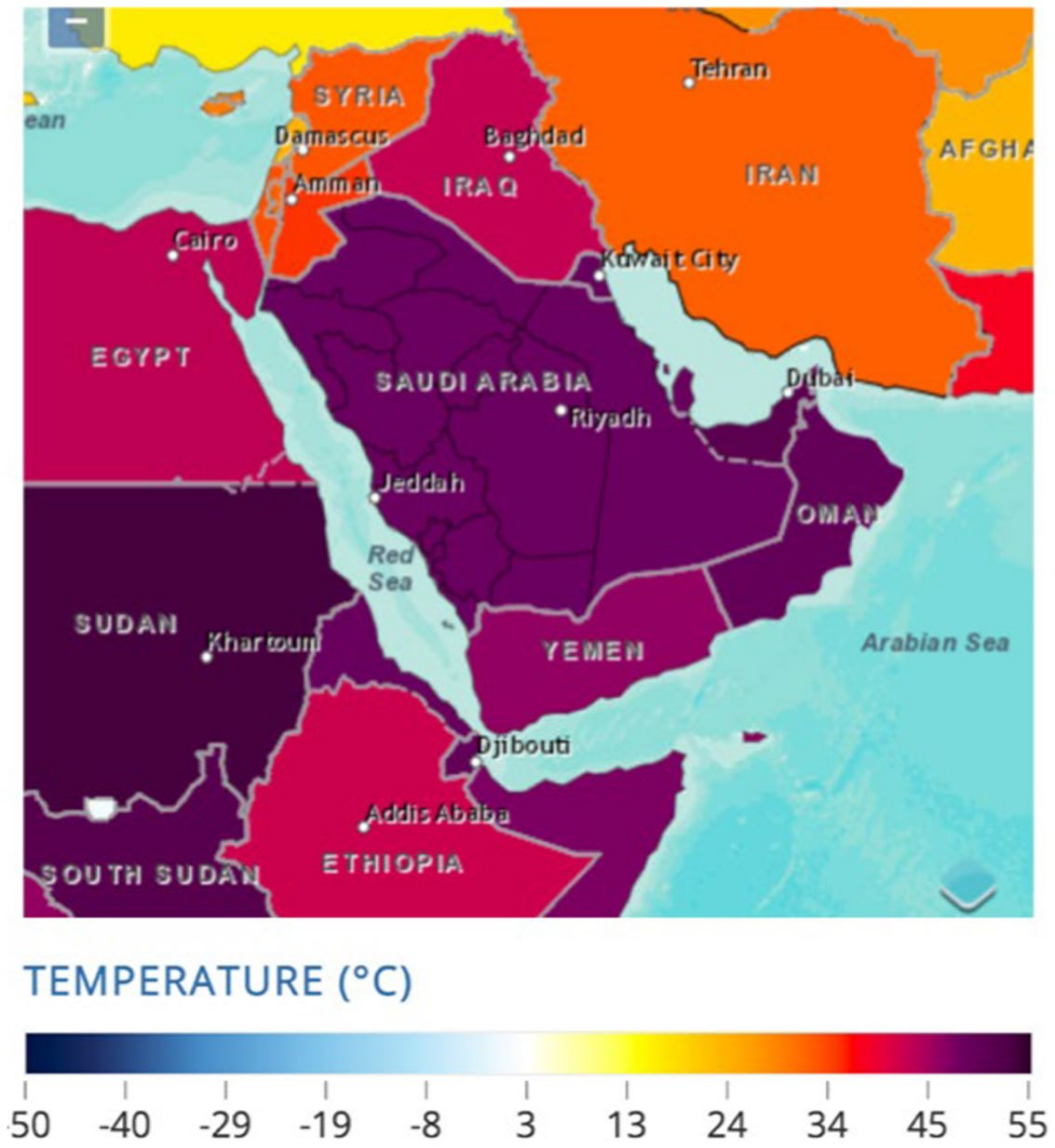
Observed annual mean-temperature 1991–2020 Saudi Arabia. Source: World Bank (7).

Saudi Arabia is located in southwest Asia and occupies around 45% of Arabian Peninsula. Moreover, 38% of its total area (2.25 million km^2^) are deserts. Due to topographical features of Saudi Arabia, the climate varies according to each region with a very hot summer (Figure 2). Saudi Arabia has a sensitive ecosystem with around 76% of its total area are non-arable including vast desert lands with absence of rivers and lakes. The scattered pasture lands consist only of low productivity shrubs and herbs that have manage to survive with low average of rainfall with extreme conditions (24). Accordingly, sensitive desert ecosystem, agricultural productivity and low water resources make Saudi Arabia vulnerable to climatic change (25). Therefore, climate change has shown eventual impacts on population health in Saudi Arabia ranging from loss of some healthy lifestyle habits, decrease in the production of vegetables and fresh fruits, micronutrients deficiency such as vitamin D due to inefficient exposure to sunlight (26). On the other hand, several studies revealed that climate change and extreme weather is one of the barriers that increase adopting a sedentary life style. Accordingly, in a study conducted on a representative sample of primary school children in Riyadh the capital city of Saudi Arabia, it was found that the proportion of children walking to schools was only 28.7% resulting in an 15% increase of body fat compared to students walked to schools (27). Furthermore, a recent study focused on the barriers and facilitators to exercises among Saudis adults concluded that among the barriers, hot weather ranked first at the environmental level (28). Moreover, climate change has given a risk of the emergence of infectious diseases respiratory diseases such as Middle East respiratory syndrome, noting that millions of pilgrims visit Saudi Arabia for Hajj and Umrah each year from more than 180 countries, which act as crucial factor for increasing the risk of spreading infectious diseases among the population (29). On the other hand, the result of an observational study conducted by Saber and his colleagues, showed that increased temperatures are significantly associated with the incidence of heat stroke and heat exhaustion (30).

Additionally, the effects of climate change, particularly temperature increase has been more drastic in the Mediterranean regions (31). These countries reached a temperature above average by 1.5°C which is higher than that recorded in other parts of the world which is about 1.1°C (32). Also, according to MedEcc (32), this number is estimated to increase to 2.2°C or even 3.8°C by 2040, and these elevated temperatures could potentially cause an increase of intensity and frequency of heat waves, a decrease in precipitation, an increase in torrential rain or extremely heavy rain fall, scarcity of water, and an effect on agriculture and fishing. On the other hand, Lebanon is a country in West Asia, in the middle east. The country is known for its Mediterranean type of climate. But the annual average mean surface air temperature of the country has significantly increased from 14.25°C in 1901 to 15.66°C in 2021 (7). Another aspect of the environmental sector which is affected by climate change is the availability of water resources. In Lebanon, for instance, suffers from the poor management of water resources and their transportation to houses, farmlands, and factories (33). Also, as reported by Halawni and Halawani (33), this unavailability of water sources could potentially lead to significant decrease of water reserves available in lakes, aquifers, and reservoirs or hydrologic drought. In addition to that, it’s been estimated by The Ministry of Environment that Lebanon will potentially experience a decrease of 14% in its GDP by 2040 and this percentage will increase to 32% by 2080 (34). Moreover, with COVID-19 spreading and the August 2020 Beirut port explosion, citizen’s health has deteriorated, this explosion resulted in the production of NO_2_ in the atmosphere. A study conducted by showed that the exposure to high levels of NO_2_ can have a negative impact on health such as correlation with high blood pressure, association to cardiovascular diseases in addition to the occurrence of obstructive pulmonary disease (35). Other references showed also the impact of GAZA war on Lebanon’s environment. The UNDP report shows the use of white phosphorus with other factors have caused extensive environmental harm, impacting natural ecosystems, water quality, and posing ongoing risks to human health and safety (36) and the UN Resident and Humanitarian Coordinator for Lebanon expects their health to deteriorate even further as a result of climate change (34).

#### 3.1.2 Global climate change challenges

**Figure 3:**
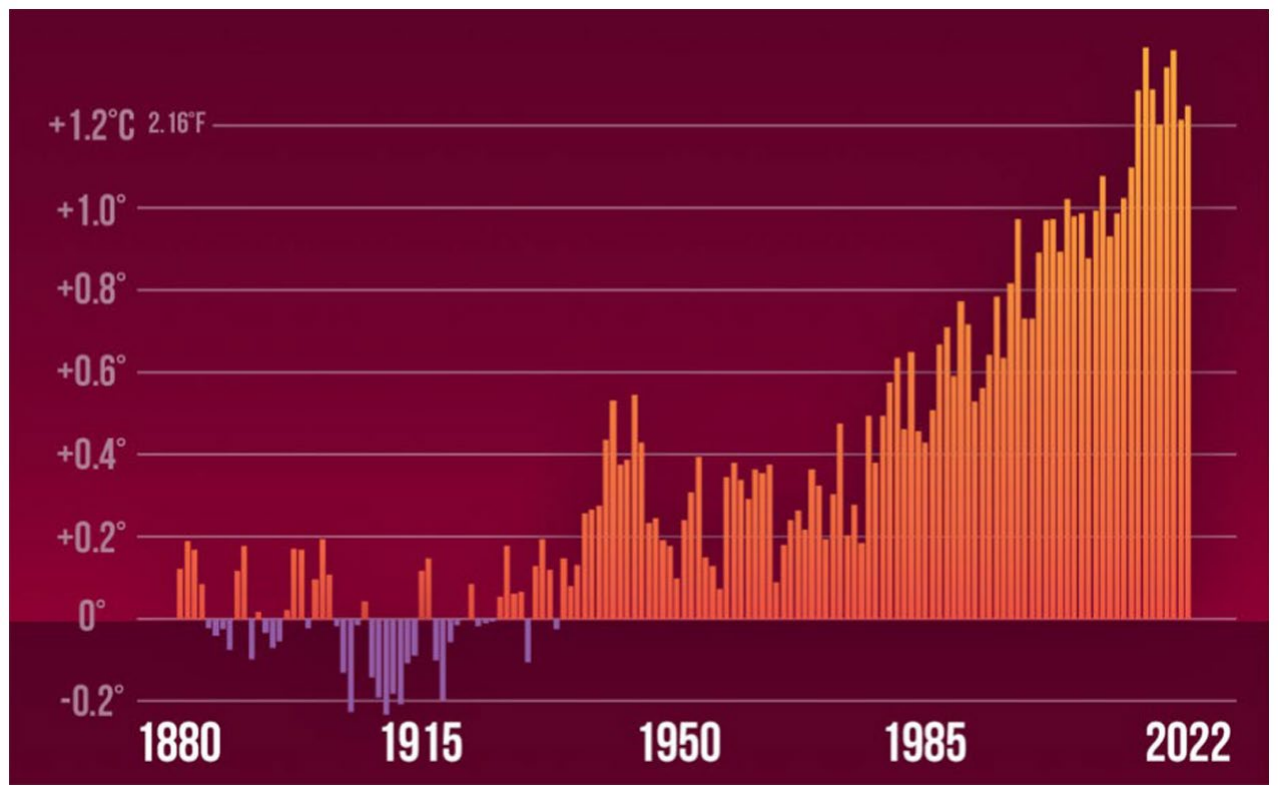
Global average temperature anomalies, departure from 1881 to 1910. Source: World Bank (7).

During 2021 and 2022, more pressure was added by the extreme weather conditions to world’s population health and health services which are still struggling with the impact of COVID-19 pandemic, worsening the human health situations, especially for the most vulnerable population. Climate change has been identified as the greatest health challenge of the twenty-first century (37). It is bringing more deadly extreme heat and wildfires, increasing non-communicable diseases and facilitating the emergence and spread of infectious diseases, contributing to health emergencies (38). Wildfires in Huawaii, Canada, Algeria and Turkey; floods in China, Malaysia and South Sudan resulted in thousands of deaths and injuries, millions of people have been displaced beside the economic losses estimated by billions of dollars ((7);). Moreover, recent studies have quantified the influence of climate change with its different aspects, where the global average temperature has increased around 1°C since 1880 (Figure 3), vulnerable population including people aged more than 65 years and children less than 1 year showed an increase in heat-related deaths by 68% in 2000–2004 and 2017–2021 (39). Concurrently, climate change is putting populations at risk of the spread of infectious diseases such as malaria and dengue fever. Interestingly, in South America, Asia and Africa. COVID-19 pandemic worsens the health situation and exhausted the healthcare systems in the management of the pandemic due to misdiagnosis between the coexistence dengue fever along with COVID-19 (38). Therefore, the relationship between national management practices and global climate policies is considered as intricate, which will be facilitated through cooperation between cities and pushed by international agreements (40).

### 3.2 Health impact of climate change in Saudi Arabia and Lebanon

Saudi Arabia was ranked by The Global Climate Risk Index 2018 as the 84th country adversely affected by climate change (41). The nation’s per capita energy consumption and CO_2_ emission are among the highest globally. In 2014, Saudi Arabia consumed around 4 times the world average oil with enormous CO_2_ emission rendering this country in the seventh place globally CO_2_ for per capita (42). As well as in Lebanon, in accordance to Dr. Iman Shankiti’s, the representative of the World Health Organization in Lebanon, speech, approximately 1million people die each year as a result of living and working in unhealthy environments. Shankiti also mentioned that about 23% of disease cases, each year are caused by environmental causes. Most importantly, children represent 30% of the population affected by the adverse effects of climate change. Lebanese people usually depend on generator powered electricity, the fumes released by burning fuels to generate these machines ultimately cause an increase in air pollution, hence affecting the health of the people who breathe this polluted air (22).

Accordingly, Saudi Arabia and Lebanon are among the global countries that face sever climate change challenges with negative impact on human health such as:

#### 3.2.1 Food insecurity

**Figure 4:**
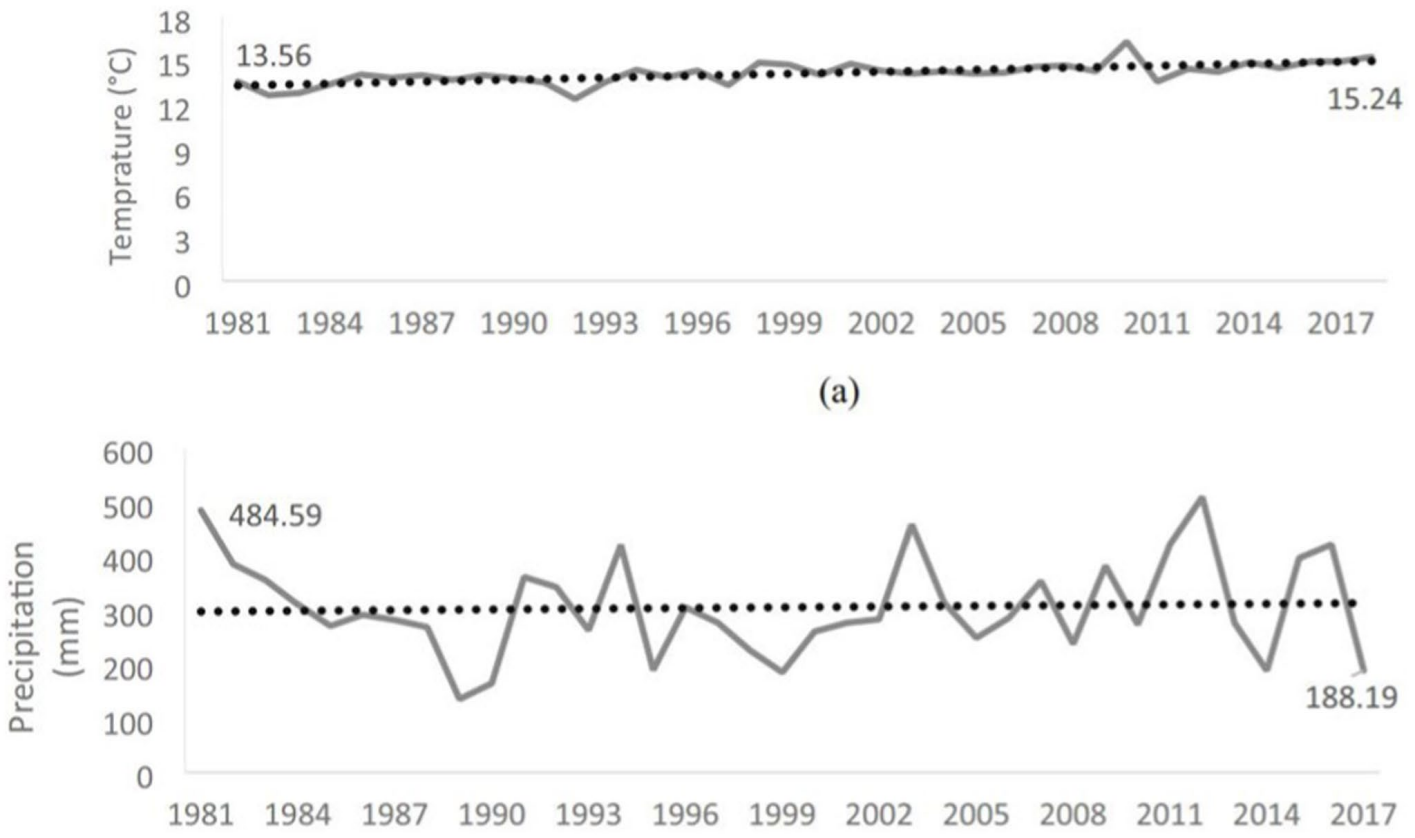
**(A)** Average annual temperatures (°C) of the Bekaa Valley (at 2 m) from NASA POWER data (1981–2018) (https://power.larc.nasa.gov/). **(B)** Average annual precipitation (mm) of the Bekaa Valley from NASA POWER data (1981–2017) (https://power.larc.nasa.gov/).

Food security is interchangeable with the food production process. Therefore, climate change threatens food security by influencing food availability, access, supply and food price stability (43). When it comes to Saudi Arabia, there is enough evidence from the literature that climate change resulted in the loss in crop yields, where Haque et al., indicated that a one-degree Celsius increase in temperature reduces crop yields by 7–25% (44). Moreover, low water availability, soil salinization and hot seasons worsen the situation (45). Recent study has shown that in the last 50 years the average temperature has been increased by 1.9°C and the precipitation has decreased resulting in droughts, water bodies drying and ecosystem degradation that adversely affected agriculture crops (44). A significant decline in wheat crops, barley, sorghum as well as dates production. Accordingly, the estimated loss over the periods in all the crops ranges from 7 to 25% (44). When it comes to Lebanon, the agriculture is a sector heavily related to causing climate change, as anthropogenic greenhouse gases are released as a result of agriculture, in turn climate change heavily impacts the process of agriculture (46, 47). Every crop requires certain environments from temperature of water levels and sunlight exposure to grow properly. The increasingly discernible exacerbation of climate change caused an irregular pattern of precipitation and unusually high temperatures, which impacted the water accessibility, decreased the growth of coveted crops, and increased the growth of weeds and pests (48, 49). Farmers in developing countries rely heavily on rain for the crop’ growth hence they are heavily impacted by (47, 49, 50). In Lebanon, a study was done by Al Dirani et al. (51) to examine food security in small family farmers in Bekaa Valley with relevance to climate change. An overall increase in temperatures and a decrease in precipitation was recorded (Figure 4). They found out that among these families only 7.5% of them are food secure while 89% fail to achieve food security according to the Household Food Insecurity Access Scale (HFIAS).

#### 3.2.2 Road traffic accidents

Beside the discussed change in the climate of Saudi Arabia and Lebanon in terms of temperature, Saudi Arabia experiencing variations of dust storms, and rainfalls rates (52). Recently, Tarawneh and Chowdhury (25) have shown a decrease of rainfall in many regions in Saudi Arabia with several sand storms over the year, the eastern part of Saudi Arabia experiences the highest number of dust storms from10 to 60 events yearly. The already mentioned hazardous events attributed to climate change are among causes of road crashes beside other factors, such as the use of mobile, age, gender, emotional situations. A recent study has shown that the average temperature (ATM), rainfall (ARF), frequency of sandstorms (FST) was significantly associated with the total number of accidents (TAC) (Table 1). Moreover, these results showed the TAC was significantly associated with the total number of injuries (TIJ) and deaths (TOD) in Saudi Arabia (Table 2). These findings were in accordance with (53–55) showing that temperature, rainfall, sandstorms, snow and number of vehicles were responsible for the increase in the rate of injuries and deaths due to road accidents. According to the World Health Organization (WHO), road accidents have a high cost in terms of lives, which is equal to about 1.25 million people annually (56).

**Table 1:** Association of inside-city accidents (ICA), outside-city accidents (OCA), and total accidents (TAC) with climate events in Saudi Arabia.

Variables	Model 1: (POLS) DV = ICA	Model 2: (POLS) DV = OCA	Model 3: (POLS) DV = TAC
ATM	4691.04 ***	688.86 ***	5379.91 ***
	(−931.59)	(−163.32)	(−1000.09)
ARF	6960.86 ***	1343.43 ***	8304.3 ***
	(−1567.65)	(−274.83)	(−1682.91)
FST	1610.9 **	22.69	1633.6 ***
	(−759.14)	(−133.09)	(−814.95)
TVH	0.086 ***	−0.012	0.099 **
	(−0.035)	(−0.006)	(−0.038)
Constant	474.4	31.73	306.1

The values in parentheses represent the error; the symbols, * stand for significant probability value at *p* < 0.05 and <0.01, respectively. Source: Islam et al. (52). Available from https://www.mdpi.com/2225-1154/7/9/103.

**Table 2:** Association of total number of injuries (TIJ) and total number of deaths (TOD) with inside-city accidents (ICA), outside-city accidents (OCA) and total accidents (TAC) in Saudi Arabia.

Variables	Model 4: (RE) DV = TIJ	Model 5: (FE) DV = TIJ	Model 6: (FE) DV = TOD	Model 7: (FE) DV = TOD
TIJ			0.004 **	0.004 **
			(−0.002)	(−0.001)
ICA	0.011 **		0.0006 ***	
	(−0.005)		(−0.0001)	
OCA	0.124 ***		0.001	
	(−0.028)		(−0.0007)	
TAC		0.017 **		0.0006 ***
		(−0.005)		(−0.00001)
Constant	1883.1	2283.3	661.89	662.33

The values in parentheses represent the error; the symbols, * stand for significant probability value at *p* < 0.05 and <0.01, respectively. Source: Islam et al. (52). Available from https://www.mdpi.com/2225-1154/7/9/103.

Similarly in Lebanon, according to the WHO (2023), approximately 1.3 million people die as a result of road traffic accidents and 20–50 million people suffer from other injuries. Furthermore, the International Committee of the Red Cross stated that storm surges, flooding, and erosion will increase as a result of a 30–60 cm sea level rise by 2050. The extreme weather that results from climate change is usually a leading cause for road traffic accidents in Lebanon (57). For instance, the accidents connected to high temperatures showed a significant increase between 1990 and 2019 (58).

#### 3.2.3 Emergence of infectious diseases

The majority of infectious diseases are greatly affected by the variability of the climate (59) where the change in rainfall pattern with increased temperature create a favorable environmental condition for pathogenic microorganisms as well as vector-borne diseases (60). Interestingly, food-, water and vector-borne diseases arise significantly in vulnerable regions such as the developing countries of the Easter Mediterranean and Middle East (EMME) countries such as in Saudi Arabia. According to WHO, climate change contributes to increasing emergence of infectious agents with low disease control, as illustrated by the spread of COVID-19 pandemic in many countries of EMME (61). Accordingly, the study performed in Jeddah, one of the largest cities in the kingdom, has shown that the dengue seasonality was significantly associated with the increase in temperature and decrease in relative humidity (62). Although the recent World Malaria Report declared that many countries of EMME including Saudi Arabia have achieved their target for malaria-free country, malaria vectors represent a threat highly sensitive to the changes affecting the climate of Saudi Arabia (12). Therefore, climate patters and influx of immigrant workers from endemic countries as well as the huge number of pilgrims visiting Holy cities yearly will increase the burden of climate change-induced malaria on healthcare systems. These findings were in accordance with Salimi and Al-Ghamdi (63) showing that an increase in 1°C of temperature caused a significant increase (15–25%) in malaria incidence. Moreover, climate change increases the risks of food-borne diseases. Al‐Rifai et al., (91) reported that the incidence of non-typhoidal *Salmonella* cases and other food-borne illness has increased in the last decades, since the survival and multiplication of food-borne pathogens in water and food is greatly affected by increased temperature posing Saudi population’s health at risk especially the most vulnerable people. When it comes to Lebanon, the change in temperature and precipitation patterns caused an increase in pathogenic microorganisms and vector borne diseases emergence (59, 60). The developing countries of the Easter Mediterranean and Middle East (EMME) countries including Lebanon have shown vulnerability to the rise in food-borne and vector-borne diseases. According to WHO, pathogens that cause diseases with low disease control are more likely to spread which was the main reason for the COVID-19 spread. Lebanon is already facing an upsurge of infectious diseases for various reasons like the Beirut port explosion. Climate change is an additional cause that has been contributing to this increase in infectious disease appearances. An example to that would be the cholera outbreak Lebanon faced which started on the 6th of October 2022 for the first time in 30 years. As per Venkatesan 2023, on the 26 of November, 4,337 cases of suspected and confirmed cholera had been reported along with 20 associated deaths, resulting in a case fatality ratio of 0.46% (64). Other types of infectious pathogens have started abruptly spreading such as Ae. albopictus and non-typhoidal Salmonella in multiple countries of The Eastern Mediterranean and Middle East (EMME) region including Lebanon (65). By analyzing the results of observational data sets from Turkey, Cyprus, and Greece, a correlation was made between the elevated temperatures, and sandfly abundance hence and spread of vector borne infectious diseases (66).

### 3.3 Future adverse impacts of climate change on health in Saudi Arabia and Lebanon

The projected future temperature in Saudi Arabia showed an increase exceeding the threshold and will record average temperate unsuitable for human (7). Saudi Arabia has warmed at a 50% higher rate than the rest of the landmass in the Northern Hemisphere. The expected increase in temperature in the future will significantly affect health resulting in heat-related mortality extreme weather events and thermal stress linked to climate change (67). The number of heat-related deaths in the Kingdome has increased by 89% between 2000 and 2004 when compared to the period between 1990 and 2018, most deaths accounted for labors, thus total labor is expected to decline by 12.3 and 22.1% under a low and medium CO_2_ emissions scenario, respectively. A recent study showed that the average annual heat-related death rate across MENA countries is currently 2·1/100,000 people the. In Saudi Arabia, the total annual heat-related deaths recorded 285 cases and the annual heat-related mortality rate is 1.03/100,000 people. Moreover, Arab countries in the Persian Gulf will be experiencing the greatest relative annual increases in heat waves and mortality related cases. For instance, by 2061–80, Saudi Arabia even under a low emissions scenario will show 13-fold increase in heat-related death rates (68).

**Figure 5:**
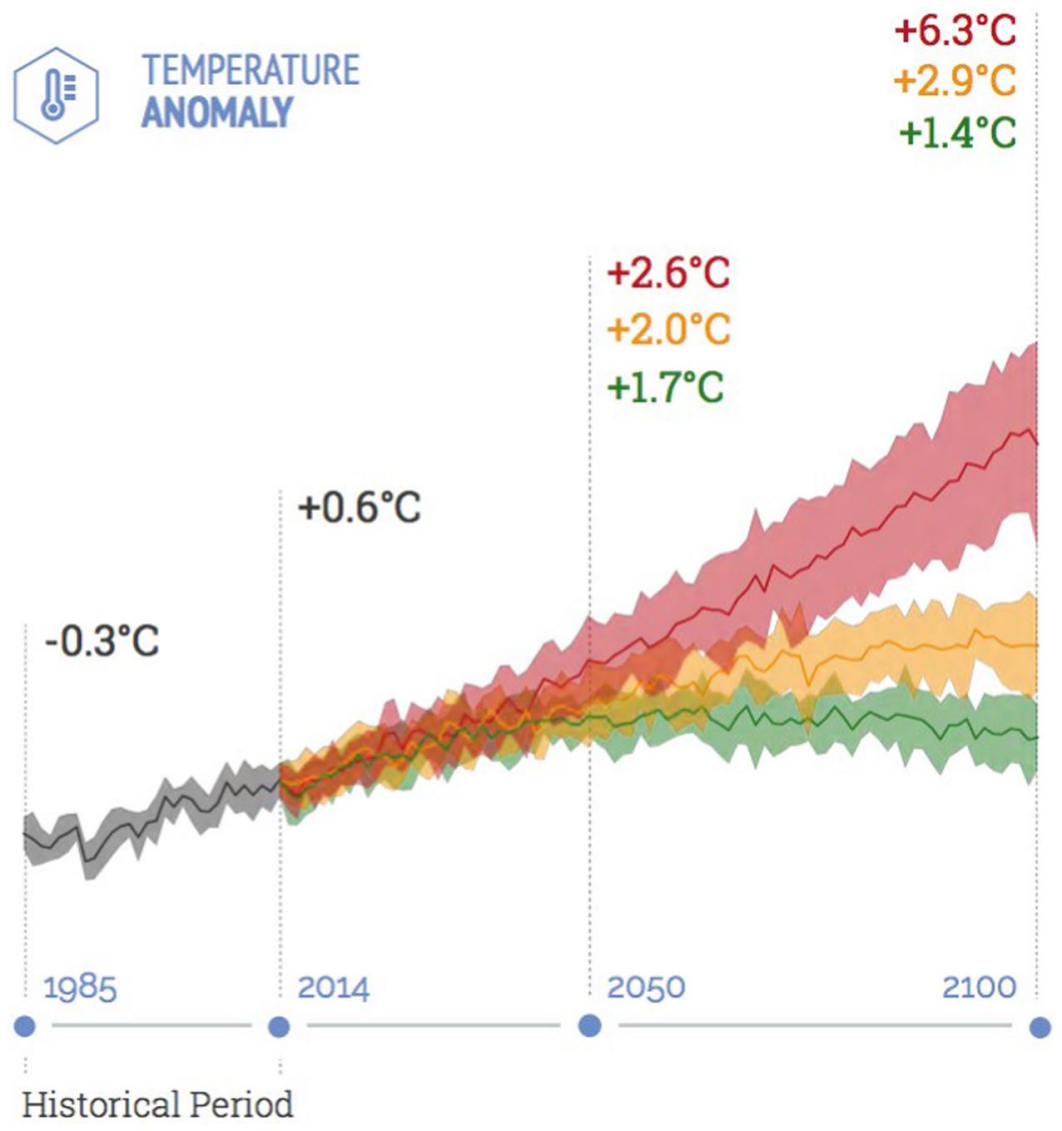
Temperature projection in Saudi Arabia as indicator of climate change. Source: CMCC, 2021. Available from https://www.g20climaterisks.org/saudi-arabia/

Premature mortality due to pollution is another aspect of health threats since in Saudi Arabia in 2017 around 315,200 disability-adjusted life years (DALY’s) were attributable to fine particle matter pollution (PM 2.5). On the other hand, (69) declared that Saudi Arabia is among the most adversely affected countries by climate change. Therefore, the estimated loss of crops will increase by more than 5% in 2099 as projection of mean global warming of 2.9°C by 2,100 (Figure 5).

**Figure 6:**
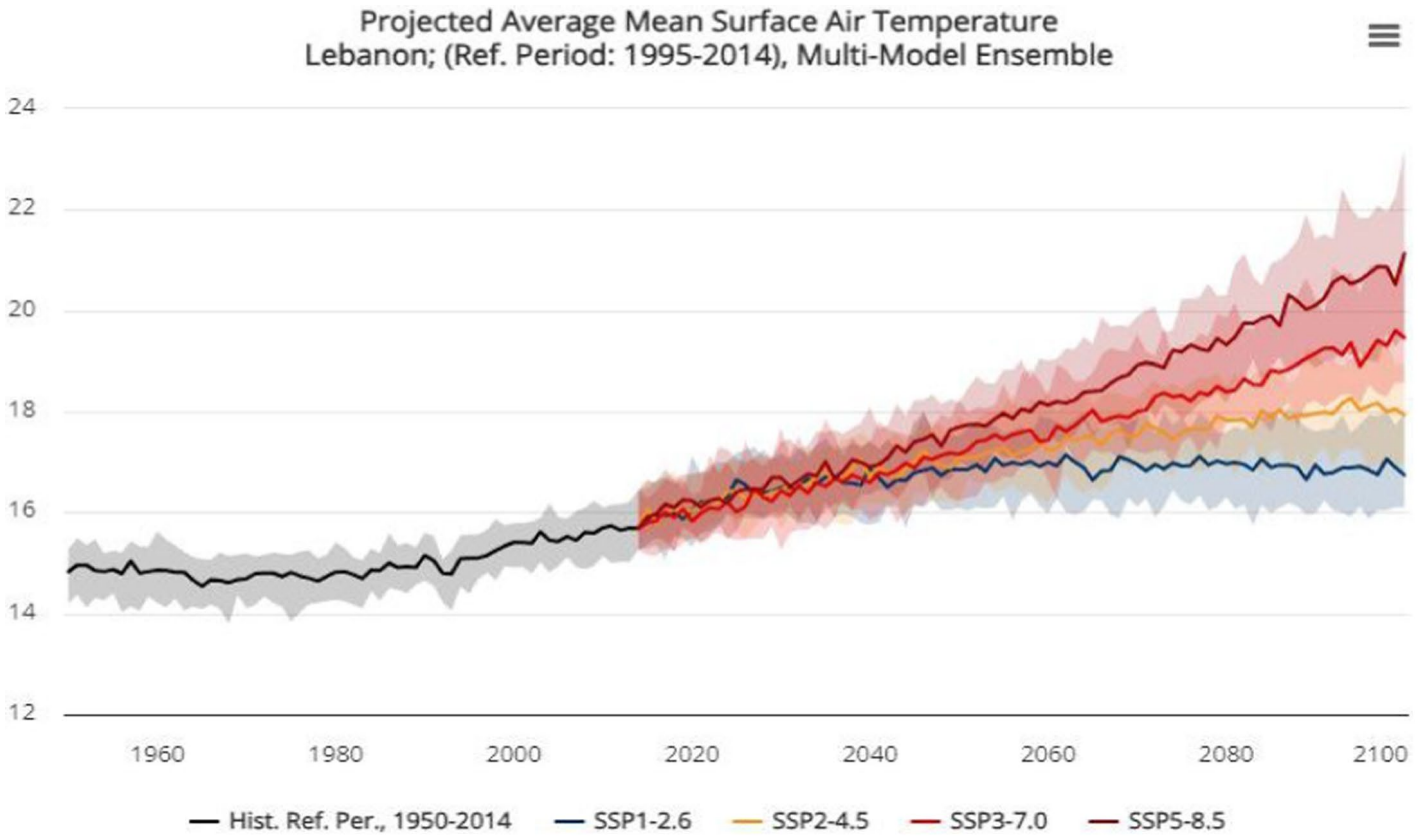
The development of the average mean surface air temperature in Lebanon.

Moreover, PRECIS climate model and DSAT models for prediction of crop production showed that the Kingdome is expected to experience a significant decline in agriculture productivity by 2050. Demand on Oil is another story, (70) declared that oil demand declines quite dramatically as temperatures rise. Therefore, the decrease in oil demand will lower its price nearly 14% by 2048. This would create a great deal of economic stress in Saudi Arabia where the economy will decrease around 10% by 2048. In like manner, in Lebanon: Najat Rochdi, the UN Resident and Humanitarian Coordinator for Lebanon, believes that as time goes by, climate change effects will prove to be extremely dire on the economic sector, citizens’ livelihoods, their health, and the availability of natural resources (71). In fact, the World Bank estimated the potential effects in the upcoming years in accordance with data analysis and modeling strategies. For instance, Lebanon is expected to experience a significant rise in the average mean surface air temperature by 2080–2099 (Figure 6). Also, a decrease in the number of days where the temperature reaches below 0°C would be anticipated by the year 2040–2059 which results in milder winters. Precipitation is also expected to increase with up to 99% which results in irregular irrigation for the agricultural sector. Furthermore, people’s health is expected to face detrimental consequences in the future with the expectations of the further increase of food borne, vector borne, and water borne pathogens especially in the summer. Because of electricity shortages and the inability to refrigerate food in the temperatures necessary to keep them edible, the probability for these foods to be contaminated increase (60). Also, a high predicted probability for infections in the eastern Mediterranean coast with West Nile Virus has been found by 2025–2050 in a study conducted by Semenza et al. (72). An increase in infections with Leishmania and Phlebotomine is expected in the Middle East (73).

### 3.4 Proactive measures to mitigate climate change

#### 3.4.1 Effective actions to address climate change challenges in Saudi Arabia

Al-Ahsa was selected by the United Nations Educational, Scientific and Cultural Organization (UNESCO) as a World Heritage Site in 2018 since it is the oldest region in the world evolving the biggest oasis worldwide (74). Al-Ahsa Oasis, shares borders with United Arab Emirates, Kuwait, Bahrain, Qatar and Oman covering an area of 2,500 Km^2^ in the Southern part of the Eastern Region of Saudi Arabia. It has a hot and dry summer and a moderate to warm winter with occasional showers and high frequency of dust/sandstorms among all regions in the kingdom (52). According to the housing census of 2016, the population of Al-Ahsa has a current population estimate of 768,000. According to the New Urban Agenda (NUA), Al-Ahsa needs to achieve environmental sustainability by mitigating and adapting to climate change since it recorded the highest temperature in the Kingdom accompanied with frequent sandstorms (74). Mitigation of local effect of climate change could be achieved through 3 different actions:

##### 3.4.1.1 Agriculture and food security

Al-Ahsa is strongly rooted in its agricultural heritage, but trying to balance its heritage with climate change is a big challenge to ensure food security (75). Therefore, the implementation of sustainable agriculture techniques that aim to reduce water consumption and food waste is essential. Vertical agriculture is a strategy that allow to grow crop with less water, less herbicides and pesticides use. Hydroponic method is one type of vertical agriculture that uses water to grow plants instead of soil, indoors and in any climate (76). Importantly, UAE the neighboring country to Al-Ahsa, as it has desert environment of the Arabian Gulf region, should prioritize sustainable agricultural and consumption practices, thus it adopted in 2018 a new climate-smart farming techniques as one action of UAE’s National Food Security Strategy 2051. These strategies promote water conservation, reduction of food waste and increasing productivity. Therefore, recently UAE built the world’s largest vertical farming that use less water than outdoors regular agricultural lands by 99%. Consequently, food waste has decreased significantly by adopting such climate-smart agricultural methods, and by 2030 UAE is aiming to cut food waste effectively (77).

##### 3.4.1.2 Use of renewable energy

There is many evidence on the realities of climate change ranging from increase in sea level due to glaciers melting to heat waves as well as hazardous snow and sand storms. Therefore, it is about time that nations adopt renewable energies sources to control their emission of greenhouse gases as mitigation strategies of climate change challenges (78). Accordingly, the renewable sources of energy include sun, wind and water. However, this action of transmission to renewable energy has some limitations since not all countries receive the same amount of solar energy, the economic cost as well as other environmental factors (79). However, Al-Ahsa is receiving high solar radiation over the year, unfortunately not switching to such green energy resides to two main root causes. Firstly, the low price and availability of fuel in Saudi Arabia make the production of energy by fossil burning more affordable and easier to be accessed. Secondly, solar energy system generates energy with low efficiency and not reliable for constant and continuous productivity. Nevertheless, the grid integration to electrical supply affects significantly the amount of greenhouse gases emission. These findings are in accordance with Qatar State vision, which is a Gulf Cooperation Council with abundant oil resources rendering it an energy secure country. However, Qatar has adopted the integration of renewable energy in electricity production, researches declared that this integration decreased emission of CO_2_ beside an increase in hydrocarbons for export (80).

##### 3.4.1.3 Awareness campaigns on climate change and health

Reducing Greenhouse gases emission through societal support and behavioral changes could be enhanced by increasing population’s awareness toward climate change impact on health (22). Recruiting healthcare professionals and health promotion specialists may facilitate the implementation of actions planed by the awareness campaigns for climate change adaptation and mitigation that decrease population vulnerability and promote health. The aim of such type of campaigns is to: (i) share information with audience usually interested in health without taking into consideration the climate change that impact it; (ii) address the human made activity contributing to climate changes; (iii) Emphasis the importance of green space as natural sink of CO_2_; move from interest to action phase by providing audience with material, tools and manuals to understand the health impact of climate change. Accordingly, Increasing the public awareness toward the adverse impact of climate change on health was adopted by the Ontario Public Health Association by developing a communication strategy in terms of increasing partnership between environmental sectors, community and health professionals. In 2018, The Atmospheric Fund funded this initiative which emphasis evidence-informed health-climate project (81).

#### 3.4.2 Strategies for combating climate change effects in Lebanon

The Bekaa Valley, is a fertile agricultural land in Lebanon, located between the Mount Lebanon and Anti-Lebanon mountain. It spreads for 120 kilometers (75 miles) in length. The land is known for its agricultural activities, including the farming of various crops and vineyards. It is a vital agricultural and economic source in Lebanon as it contributes to the country’s agricultural production and economy. The valley is also historically and culturally rich, with archaeological sites dating back to ancient civilizations (82). Just like any other agricultural land, this land is also facing the consequences of climate change for instance, Abdel-Wahab Amhaz, head of a Shia Muslim clan in Nabha, in northern Bekaa said in a statement recorded by The New Humanitarian newspaper “Nothing’s profitable to grow here. Twenty thousand dunums (nearly 5,000 acres) of farmland is unused,.” Because of its significant impact on Lebanon’s agricultural and economic sectors, it’s vital to find solutions to lessen the intensity of the impact of climate change on the valley.

##### 3.4.2.1 Agriculture and food security

In order to mitigate the intensity of the impact of climate change on agriculture, an application by Baban (83) was done using satellite remote sensing and GIS (Geographic Information Systems) technologies mainly to manage water sources in the Middle East. This application was used to provide management for water quantity and quality, sedimentation and floods, round water and soil moisture, and land use and crop monitoring. The main purpose for this study is the understanding of available water sources in order to be able to integrate them into potential solutions to solve water scarcity which has been lately in the Middle East including Lebanon. In addition to that, trying to prevent the contamination of fresh water with sea water along with working toward enhancing the quality of water sued in homes, industries, and farms, would help in decreasing the impact of climate change on clean water availability. Moreover, in order to be able to control the issue of food insecurity certain actions should be taken like enhancing irrigation systems, forming new types of plants that can survive in the new environmental conditions, and changing the farming schedule including irrigation and harvesting to fit these conditions.

##### 3.4.2.2 Use of renewable energy

To reduce greenhouse gas release and accumulation, nations should take into consideration replacing their sources of energy (78). Some of the natural resources that can be used in order to decrease the effect of fumes released by burning fuels would be the sun, water, and wind. Unfortunately, the availability of these resources also varies from country to another (79). In Lebanon, the dominating source of energy besides burning fuels is hydropower, where it has 5 hydroelectric power plants (Table 1) distributed from south to north specifically in Western Mount Lebanon. Hydropower is a suitable The Litani River is the longest flowing river in Lebanon; it rises in the Beqaa valley and empties in the Mediterranean Sea in Saida, it has an annual flow of 920 million cubic meters a year. This makes it a perfect location for hydroelectric plants hence making the Litani power plant the most reliable hydropower source in Lebanon. The Litani River Dam was built in 1959 as a way to generate electricity. Although there’s no specific farm for windmills in Lebanon, there exists a few wind turbines that are used for personal use or for private factories. Furthermore, solar energy is prevalent in Lebanon where 5.01 kWh/m2/day of solar radiation (84).

##### 3.4.2.3 Awareness campaigns on climate change and health

The WHO (2023) has collaborated with several organizations like the European Commission and the European Environment Agency to help the Health Observatory raise awareness on the relationship between climate change and health. It also emphasized the importance of spreading information about greenhouse gases’ effects on climate change and health. Awareness campaigns usually would grab the attention of those interested in enhancing human health and aren’t aware of the huge impact climate change has on human health. Recruiting healthcare professionals and advocates would help promote the initiatives taken by advocates to overcome the health issues resulting from climate change. These campaigns aim for making this connection between human and environment health clear and spread information about the harmful activities humans act upon which might serve as detrimental for the environment. They also provide evidence to people on how harmful greenhouse emissions actually are and motivate people into opting to act for the environment. In 2018, The Atmospheric Fund funded the Ontario Public Health Association which aimed to increase social awareness to the impact of climate change on health by enclosing the distance between health professionals, the ecological sector, and people (81).

### 3.5 Climate change fund: pros and cons

The main objective of applying for funds to mitigate climate change is to support and promote initiatives and activities that focus on increasing awareness among public through case studies documentations and left behind brochures, sharing knowledge between health professionals, population and decision-makers via workshops and conferences, supporting sustainable development goals (SDGs) projects. On the other hand, there are three main types of funding methods namely Non-Governmental Organizations (NGOs), Governmental ministries and International Organizations.

#### 3.5.1 State government funds

State of the government funding is a type of funding which covers the expenses for several types of activities that aim to benefit the community. Usually, each government is supposed to award fund to activities and initiatives related to its priorities, responsibilities and objectives. For example, in Al-Ahsa, The Ministry of municipal and Rural Affairs (MoMRA) has the role of supporting municipal activities including infrastructure, irrigation channels and road maintenance, urban planning (74). However, schools service and needs at Al-Ahsa are the responsibility of the Ministry of Education not the MoMRA. Applying for governmental grants has many advantages. Firstly, the most valuable benefit is that you are not supposed to pay back what you have received from the government, eventually this occurs in case of funding project of great impact on community and local region. For instance, in Lebanon, this category of funding is characterized by its non-repayable nature, signifying that recipients are exempt from the obligation of reimbursement. Also, this funding can be used for several types of projects like healthcare, economic, education projects (85). Lastly, it is noteworthy to emphasize that projects funded by local governmental entities hold a heightened appeal for stakeholders, consequently enhancing the project’s credibility and likelihood of success.

Finally, it is worth mentioning that project funded by local government are more attractive for stakeholders which increases project’s credibility and success. Although governments grants have many benefits, some disadvantages have to be discussed. Firstly, applying for such funding methods has a complicated process and needs evidence for the project’s worth. Secondly, the process may take several months to be approved or rejected. Thirdly, the government usually accept supporting projects that meet with its needs and aligns its mission, which may decrease the possibility to get approved for fund.

#### 3.5.2 Non-governmental organizations (NGOs)

According to the World Bank, NGOs as organizations that accept low interests, support community growth with skilled persons and technicians who implement their objectives effectively (86). When it comes to the advantages of NGOs, they are playing a crucial role to deliver information to communities mainly in developing countries. Since Lebanon is a developing country, numerous NGOs that opt for reducing the effects of climate change and preserving the environment exist. Some of these NGOs would be Drop-Off Tabarja which aims to sort recyclable materials. Most importantly that NGOs grants usually are awarded to support initiatives with fast remarkable impacts. For example, in India, NGOs were integrated in waste management facilities, which had increase communities’ involvement in waste management by addressing the barriers of this process (87). The most important disadvantage of NGOs is that their funds are limited in budget that support small projects.

#### 3.5.3 International organizations

Green climate fund is one example of an international organization that award fund to mitigate climate change, founded in 2010 by the UNFCCC. The awarded funds are particularly addressed to countries that are greatly affect by climate change such as the low-income countries (77). The most disadvantage on trusting such type of fund is the lack of transparency in terms of who is responsible to approve funding. When it comes to this report each proposed action has a type of fund that fit to it. The agriculture and food security as well as the use of renewable resources could be covered by government funds while awareness campaigns fit with NGOs missions. Both funds have high chance to be approved but tackling the concept of climate change impact on health at community level represents a good start.

### 3.6 Response effectiveness to mitigation initiatives

In Saudi Arabia the oil-dependent industries contribute over 70% of worldwide greenhouse gas emissions, influencing the effectiveness of climate change mitigation efforts, in addition Saudi Arabia has the largest oil reverse in rendering it one of the highest per capita GHG emission. From all what have been mentioned Saudi Arabia possesses the challenges around climate change mitigation-related initiatives (88). On the other hand, in Lebanon the effectiveness of mitigation efforts to address climate changes challenges are significantly affected by the ongoing wars and conflict in Lebanon. Repetitive wars, Syrian migration to Lebanon and Gaza war affected the country’s ability to implement climate change policies and agreements, this is mainly due to several factors. First, resources diversion, where the government spend any available financial resources on security, military priorities and post-war reconstruction. Second, the destruction of environment during war, where damaging of infrastructure (electricity grids, roads) and additional pollutant released into the water and atmosphere made hard to Lebanon to reduce its carbon footprint which worsen the impact of climate change and reduces the effectiveness of any previously implemented actions. Most importantly the displacement and refugee crisis forcing people to change settlements for new secure one and this applies to Lebanese people moving from south Lebanon to the capital and those moving from neighborhood countries to Lebanon which added more burden on its economy, violations of sustainable management practices. Therefore, the effectiveness of addressing climate changes in Lebanon is continuously affected by Lebanon political stability, international support, and a long-term focus on sustainable development.

The main findings of the global impacts of climate change, then the impacts in Mediterranean region to end up with the target countries namely Saud Arabia and Lebanon in terms of current health impact, future impact, proposed actions, funding and effectiveness are summarized in Table 3.

**Table 3:** The main findings of climate change impacts for Saudi Arabia and Lebanon.

Category	Main findings
Greenhouse gas emissions and global warming	GHG emissions have increased due to the use of fossil fuel, agriculture, and waste treatment. Since 1850, CO₂ emissions have risen, leading to an increase in the temperature of the globe.
Global effects of climate change	The rise in the temperature of the oceans, melting glaciers, rising sea levels, and more frequent natural disasters (droughts, wildfires, floods, hurricanes). Climate change also made air pollution worse, cardiovascular, and respiratory diseases.
Climate change in the Mediterranean region	Mediterranean region has warmed by 1.5°C, higher than the global average of 1.1°C.
Health impacts in Saudi Arabia and Lebanon	Climate change worsens food insecurity, road accidents, and the spread of infectious diseases. Lebanon’s reliance on fuel generators contributes to air pollution, deteriorating public health.
Future impacts	Rising temperatures and irregular precipitation will affect agriculture, water supply, and disease outbreaks. Foodborne and vector-borne diseases are expected to increase.
Proposed actions	- Agriculture and Food Security: Improve water management, irrigation systems, and crop adaptation. - Renewable Energy: Encourage solar, wind, and hydropower use. - Awareness Campaigns: Public education on the health risks of climate change.
Climate change funding	- State Government Funds: Support projects that need government back-up. - NGOs: Raise awareness but have limited budgets. - International Organizations: UN-led funds support climate-affected developing nations.
Response effectiveness	Saudi Arabia possesses the challenges around climate change mitigation-related initiatives due to its high oil reserve and its high GHG emission. Lebanon possesses the challenges around climate change mitigation-related initiatives due to continuous wars and conflicts.

## 4 Limitation of the study

1. Lack of Primary Data – Data reported in the study are taken from secondary sources and literature review as direct measurements, interviews or surveys are required for further investigations.
2. Generalization of Global Trends – Despite the fact that global climate trends are discussed, however, their effects on the studied regions were not reported due to a lack in localized climate modeling.
3. Policy and Governance –There is an essential need of an in-depth analysis of the countries’ governmental policies and regulations regarding climate change.
4. Technical Feasibility of Solutions – The study proposes renewable energy and agricultural adaptations as solutions, however, feasibility assessment in terms of cost, infrastructure, and implementation barriers is needed.

## 5 Policy recommendation

1. Strengthening Climate Governance:

- Put together a national climate change strategy that specifically explains the targets across a limited timeframe.
- Form a task force whose main role is coordinating and overseeing the jobs of ministries and sectors
- Increase the regulation and enforcement of existing environmental laws.

2. Promoting Sustainable Agriculture and Water Management:

- Implement sustainable agricultural methods which include crop rotation techniques together with reduced tillage practices and agroforestry programs.
- Establish improved water storage facilities with collection methods to support water availability during dry seasons.
- Promote the acceptance of plant varieties which demonstrate resilience to drought combined with tolerance to heat.

3. Enhancing Renewable Energy and Reducing Emissions:

- Increase spending on solar and wind energy projects to substitute fossil fuel dependence.
- Provide financial benefits to households along with businesses for adopting energy-efficient technology solutions.
- Improve the city’s public transportation to reduce environmentally harmful emissions from private automobile usage.

4. Improving Public Health Preparedness:

- Establish both heat action plans and early warning systems that help respond to climate-triggered severe weather.
- Work on improving healthcare facilities for appropriate handling of climate-related diseases including respiratory diseases and infections spread by vectors.
- Raise community awareness through campaigns on climate-related health risks and prevention strategies.

5. Securing Climate Financing and International Support:

- Increase partnerships with international organizations to obtain climate adaptation funding.
- Establish collaborative agreements between public and private entities to support projects regarding renewable energy and sustainable agriculture development.
- Support research activities to improve climate risk evaluations and drive policy development processes.

In the end, implementation of strategic policy implementations will enable Lebanon to develop better resistance against climate change while conserving its resources while safeguarding public health. Active climate action requires total cooperation among policy builders with researchers and community members to build a sustainable tomorrow.

## 6 Conclusion

The constant rise of greenhouse gas emissions is a factor that contributes to several harmful environmental impacts such as rising sea levels, more frequent and intense extreme weather vents like natural disasters, increasing health risks for populations worldwide, and most importantly climate change which causes environmental, economic, and health consequences. This had been causing unprecedented temperature increases, unpredictable precipitation patterns, and worsening water scarcity in many regions around the world. Thus, climate change serves to aggravate already vulnerable environments and pose serious threats to ecosystems, agriculture, food security, and public health in general. In agricultural regions which are dependent on stable climate conditions, climate change is a factor that causes unpredictable weather patterns, prolonged droughts, and a shift in seasonal cycles which leads to reduced crop yields, threatening global food supplies and exacerbating hunger and malnutrition. In addition to that, natural disasters had witnessed an increase in incidence and intensity as a result of climate change. The increase in environmental hazards likes wildfires, floods, and hurricanes; this not only destroy infrastructure but also displace millions of people. Not only had climate change impacted the environment but also the public health, where air pollution, rising temperatures, and changing ecosystems contribute to the spread of infectious diseases. Addressing these problems needs immediate collaborative efforts at the global, national, and local levels which is why the effort of governments, international organizations, and research institutions combined is needed to implement sustainable agricultural practices, invest in renewable energy sources, and enhance climate resilience through innovative policies and technologies. As a result, investment from various national governments and international financial institutions will be critical towards enhancing initiatives toward climate change adaptation and mitigation. In the long run, leverage of strengthened cooperation between policymakers, scientists, and communities will be crucial in establishing strong systems to promote environmental conservation, safeguard diversity, and ensure public health amid both existing and future climate hazards.

## Data Availability

The original contributions presented in the study are included in the article/supplementary material, further inquiries can be directed to the corresponding author.
